# Greedy Algorithm for Deriving Decision Rules from Decision Tree Ensembles

**DOI:** 10.3390/e27010035

**Published:** 2025-01-04

**Authors:** Evans Teiko Tetteh, Beata Zielosko

**Affiliations:** Institute of Computer Science, University of Silesia in Katowice, Bȩdzińska 39, 41-200 Sosnowiec, Poland; evans.tetteh@us.edu.pl

**Keywords:** decision trees, decision rules, greedy algorithm, length, ensemble

## Abstract

This study introduces a greedy algorithm for deriving decision rules from decision tree ensembles, targeting enhanced interpretability and generalization in distributed data environments. Decision rules, known for their transparency, provide an accessible method for knowledge extraction from data, facilitating decision-making processes across diverse fields. Traditional decision tree algorithms, such as CART and ID3, are employed to induce decision trees from bootstrapped datasets, which represent distributed data sources. Subsequently, a greedy algorithm is applied to derive decision rules that are true across multiple decision trees. Experiments are performed, taking into account knowledge representation and discovery perspectives. They show that, as the value of α, 0≤α<1, increases, shorter rules are obtained, and also it is possible to improve the classification accuracy of rule-based models.

## 1. Introduction

In a time of rapid technological advancements and data analysis developments, one of the key challenges lies not only in data collection but its effective interpretation and use. In various fields and especially in medicine, finance, or management, it is important to transform large datasets into knowledge that can be understood and used for decision-making purposes. One of the tools for transforming data into valuable knowledge is decision rules—logical structures that represent the relationships and dependencies between data features (condition attributes) and their results (decision) [1].

Decision rules are transparent and easy to interpret, which makes them a proper tool for knowledge representation. Through their use, key patterns and principles underlying decision-making processes can be identified. Compared to more complex “black boxes” such as neural networks, decision rules offer a high degree of transparency, allowing users to understand models and verify results. In this context, both the way knowledge is presented and the quantity and length of the induced rules are crucial factors [2]. This aligns with the Minimum Description Length Principle proposed by Rissanen [3].

Distributed data are an integral part of today’s information and technology systems. Their effective processing and analysis can bring many benefits to organizations, contribute to process optimization, and enable real-time decision-making. Despite the challenges of their integration and security, distributed data mining technologies are developing rapidly, contributing to the creation of increasingly sophisticated and intelligent systems, used in a wide range of fields. In Internet of Things (IoT) systems, for example, distributed data make it possible to monitor the status of equipment and machines in real time, which is crucial for optimizing production processes. In medicine, distributed data help analyze patient test results stored in different facilities, enabling more consistent and faster diagnostic decisions.

The dynamic development of technologies and the increasing amount of data generated from various sources require the development of algorithms which ensure data analysis in a distributed form [4,5]. This study addresses the problem of inducing general decision rules that are true for the largest number of decision trees in a given set *S*. General rules are defined as rules that make use of arbitrary attributes from the set of decision trees being analyzed. Inner rules correspond to paths within a decision tree that extend from the root to a terminal node. A rule *r* is considered true for a decision tree Γ from *S* if there exists an inner decision rule $r_{0}$ over *S* such that the set of elementary conditions on the left-hand side of $r_{0}$ is contained within the set of elementary conditions on the left-hand side of *r* and both rules yield the same decision on their right-hand sides.

Inducing decision rules by tracing paths from root to leaf in a single decision tree is a simple process, but generating rules from a set of decision trees and selecting the most relevant ones for a particular context is more intricate. This paper is a continuation of previous research [6] where it was proved that inducing and identifying general rules that are true for the maximum number of decision trees in *S* is an optimization NP-hard problem. Consequently, it was suggested that approximate algorithms for solving this problem should be explored. In this work, the authors present a greedy algorithm for deriving decision rules from sets of decision trees. In [6], algorithm A and heuristic $H_{1}$ were presented and compared using randomly generated binary data and decision trees constructed by the Gini index. The obtained results were considered, taking into account the knowledge representation perspective. The motivation behind using the greedy algorithm is that in the case of centralized data, it has been used in the problem of optimization of the length of decision rules, and allows the induction of enough short decision rules. In addition, previous research has shown that this algorithm, under certain assumptions regarding the NP class, can produce a solution close to that obtained by the best polynomial approximate algorithms [7]. So, the greedy strategy in this work will be used to form the conditions of the left-hand side of the decision rules induced from distributed data.

The collection of decision trees acts as distributed knowledge sources, with the objective of identifying patterns that are true across the largest number of trees in the set. These patterns are expressed as decision rules, which are a commonly used and intuitive method of representing knowledge. The innovation of the proposed approach lies in applying the greedy algorithm to distributed knowledge for the induction of decision rules.

The presented research problem has many practical applications. An example is the analysis of the medical data of a patient who has been to different hospitals. The discovery of knowledge in the form of decision rules that are true for the maximum number of trees will allow the discovery of patterns that will aim to provide doctors with a complete history of a patient’s treatment to provide better diagnosis and better alignment of therapies with global health trends. The proposed approach will also optimize the resources of medical facilities by, for example, identifying facilities that have certain equipment resources or can perform certain tests.

The extraction of decision rules from distributed datasets presents unique challenges, particularly when data subsets have overlapping but non-identical attributes and cannot be centralized due to privacy or logistical constraints. While ensemble methods such as Random Forest, XGBoost, LightGBM, and CatBoost excel in predictive accuracy, their reliance on centralized data and opaque modeling processes make them less suitable for scenarios requiring interpretability and scalability. The proposed greedy algorithm addresses these limitations by operating directly on distributed decision trees, leveraging intersecting attributes to generate compact, interpretable rules. This method is particularly advantageous in decentralized environments, such as federated systems, where transparency, scalability, and adaptability are critical. Decision rules induced from distributed data help to understand the logic behind predictions and build confidence in the model. While ensemble models are preferred for predictive tasks where accuracy is paramount, they are generally considered complicated models, especially for gradient boosting methods such as XGBoost or CatBoost, as they involve a large number of trees and complex interactions among them.

In this work, in order to obtain the distributed data, the datasets are transformed into subtables using the bootstrapping technique. This creates *k* datasets containing *m* subtables, m=5,…,10 representing distributed data sources. For each subtable belonging to the set, decision trees are generated using two attribute selection criteria: the Gini index and the CART algorithm, and the entropy measure and the ID3 algorithm. Then, for each set of decision trees, a greedy algorithm is applied with an α, 0≤α<1, which represents the threshold for including additional conditions in a decision rule, balancing the rule length and generalization. The experiments conducted enable the analysis of the results from both knowledge representation and classification perspectives. In order to induce so-called true decision rules for a maximum number of trees, it is possible to increase the α value, ensuring shorter rules with a consistently good classification quality.

The paper is structured into five sections. The introduction is followed by the Background section consisting of decision trees induction algorithms, mainly CART with the Gini index and ID3 with entropy, and popular approaches for the induction of decision rules. Section 3 presents the main notions related to the optimization of general decision rules over a set of decision trees, and the greedy algorithm for the induction of true decision rules from a set of decision trees. In Section 4, the procedure of the performed experiments and obtained results are presented. Section 5 presents the conclusions and outlines plans for future research.

## 2. Background

Decision trees and decision rules are extensively utilized approaches in machine learning for extracting interpretable models from data. These techniques are grounded in the principle of partitioning a dataset based on feature values to generate decision rules or trees that predict outcomes. They are valued for their simplicity, clarity, and ability to generate human-understandable insights from complex data.

### 2.1. Decision Trees

Decision trees are a fundamental approach frequently employed for tasks involving classification and regression. They model decision-making processes through a tree-like structure, where internal nodes represent tests on attributes, branches represent the possible outcomes of these tests, and leaf nodes correspond to class labels in classification or continuous values in regression. There are a number of decision tree algorithms like CART (Classification and Regression Trees), ID3 (Iterative Dichotomiser 3), C4.5, and CHAID (Chi-squared Automatic Interaction Detector), each designed with different approaches to selecting features and handling splits with various modifications [8,9] and applications [10,11,12]. Algorithm 1 illustrates the fundamental concept behind constructing decision trees.
**Algorithm 1** Construct decision tree.**Require:** dataset   **function** BuildFullDecisionTree(dataset)        **if** all samples in dataset have the same class **then**            **return** Leaf node with the class label        **end if**        best_feature, best_threshold ← FindBestSplit(dataset) ▹ Function to find optimal split        left_subset, right_subset ← Split(dataset, best_feature, best_threshold)        left_child ← BuildFullDecisionTree(left_subset)        right_child ← BuildFullDecisionTree(right_subset)        **return** Node with left_child, right_child, best_feature, best_threshold   **end function**

This algorithm starts by analyzing a dataset with labeled examples, aiming to divide the data into subsets based on feature values and selected splitting criteria to improve class separation. This process continues recursively for each new subset until all are either pure (containing only one class) or can no longer be split meaningfully. That is, when a subset contains examples with the same label, the algorithm designates it as a “leaf node” and ceases further splitting on that subset.

Decision trees are a hierarchical approach to decision-making, where internal nodes represent tests on features, branches represent the results of these tests, and the leaf nodes denote the predicted values or classes. These trees can be viewed as a structured set of decision rules, where each path from the root to a leaf defines a distinct rule. The flexibility of decision trees makes them applicable to both classification and regression tasks, depending on the nature of the target variable. The process of constructing decision trees is inductive in nature, where the goal is to iteratively split the dataset into homogeneous subsets based on feature values. Algorithms like CART and ID3 are widely used for this purpose, applying criteria such as Gini impurity, information gain, or entropy to identify optimal splits.

In this paper, the ID3 [13] and CART [14] algorithms are considered. Both offer different strategies for constructing decision trees, with distinctions in how they handle splits, tree pruning, and performance in classification tasks. While ID3 and its use of information gain makes it more efficient for attribute selection in classification, with CART, its binary structure and focus on minimizing impurity enable it to generalize well across both classification and regression tasks. The ability to translate decision trees into rule-based systems further adds to their utility, especially in domains requiring interpretable models, such as healthcare, finance, and legal decision-making.

CART is a prominent decision tree algorithm introduced by Breiman et al. [14]. It constructs binary trees through recursive partitioning, splitting data based on a criterion that minimizes impurity at each node. CART provides a framework for both regression and classification tasks, making it versatile in a variety of contexts. In classification tasks, CART uses the Gini impurity, while in regression tasks, it utilizes the mean squared error (MSE). CART produces strictly binary trees, simplifying the model and enhancing its computational efficiency. Gini impurity is a measure of how often a randomly selected element from the set would be incorrectly classified if it were assigned according to the distribution of labels in the subset. For a set with classes $c_{1},c_{2},\ldots ,c_{k}$ and probabilities $p(c_{i})$ for each class value, the Gini impurity is given by

$$Gini=1-\sum_{i=1}^{k}p{\left(c_{i}\right)}^{2},$$

where $p(c_{i})$ is the proportion of samples that belong to the class $c_{i}$ within the subset. The Gini impurity will be 0 if all samples belong to a single class and reaches its maximum when the classes are evenly distributed.

ID3, developed by Quinlan [13], is a seminal algorithm that constructs decision trees based on information gain, a measure derived from Shannon’s entropy [15]. Unlike CART, which produces binary trees, ID3 can create multi-way splits depending on the number of possible values for a given attribute. The algorithm recursively selects the attribute with the highest information gain at each node, prioritizing those that reduce uncertainty about the target variable the most. Entropy measures the amount of “uncertainty” or “disorder” in a subset, where a higher entropy means a more diverse set of classes. It is used to assess how well a feature separates the data. For a dataset *D* with classes $c_{1},c_{2},\ldots ,c_{k}$ and probabilities $p(c_{i})$, the entropy of the dataset *D* denoted by H(D) is calculated as

$$H\left(D\right)=-\sum_{i=1}^{k}p\left(c_{i}\right)\log_{2}p\left(c_{i}\right),$$

where we have the following:

- $p\left(c_{i}\right)=\frac{|D_{i}|}{\left|D\right|}$ is the proportion of instances in *D* that belong to class $c_{i}$, and $\log_{2}$ ensures that the entropy is measured in bits.
- $D_{i}$ is the subset of *D* that contains instances of class $c_{i}$.

Consider a feature *F* with possible values $\left\{\delta_{1},\delta_{2},\ldots ,\delta_{m}\right\}$. Splitting *D* on feature *F* divides the dataset into subsets $D_{1},D_{2},\ldots ,D_{m}$ where each subset $D_{j}$ contains the instances in *D* such that $F=\delta_{j}$. The entropy of each subset $D_{j}$ is given by

$$H\left(D_{j}\right)=-\sum_{i=1}^{k}p\left(c_{ij}\right)\log_{2}p\left(c_{ij}\right).$$

The weighted entropy of the split on feature *F*, denoted as H(D|F), is the sum of the entropies of each subset $D_{j}$, weighted by the proportion of instances in each subset:

$$H\left(D|F\right)=-\sum_{j=1}^{m}\frac{|D_{j}|}{\left|D\right|}H\left(D_{j}\right).$$

The information gain shows how much the impurity is reduced after splitting. A higher information gain means that a split on a particular feature creates purer child nodes, which helps to decide the best feature to split on. The information gain of splitting on feature *F* is then the difference between the entropy of the parent node H(D) and the weighted entropy after the split H(D|F):

IG(D,F)=H(D)−H(D|F).

Substituting,

$$IG\left(D,F\right)=-\sum_{i=1}^{k}p\left(c_{i}\right)\log_{2}p\left(c_{i}\right)-\sum_{j=1}^{m}\frac{|D_{j}|}{\left|D\right|}\left(\sum_{i=1}^{k}p\left(c_{ij}\right)\log_{2}p\left(c_{ij}\right)\right).$$

### 2.2. Induction of Decision Rules

Decision rules can either be manually crafted by experts or automatically induced from data using algorithms that prioritize both predictive accuracy and model simplicity. Manually crafted rules are often derived from domain knowledge, where specialists define specific criteria based on empirical observations [16]. This approach ensures interpretability but may be limited by human biases and time constraints. On the other hand, data-driven induction methods, both direct and indirect, automate rule generation. Direct induction algorithms [7,17,18,19,20], aim to extract rules in a straightforward manner by iteratively optimizing classification outcomes, focusing on minimizing rule complexity and maximizing predictive accuracy. Indirect methods, such as those using decision tree algorithms, first construct a decision tree and then extract decision rules from its branches. These rules capture the structure of the data while simplifying complex tree models into human-readable forms. Both approaches help balance the trade-offs between model accuracy, interpretability, and complexity in rule-based learning.

In distributed data environments, the induction of decision rules from sets of decision trees becomes a critical technique for creating interpretable, accurate models across data sources without centralizing the data. Distributed data often originate from multiple locations or systems, each containing portions of the dataset with potentially varying attributes. By training separate decision trees on each subset of the distributed data, it is possible to generate a collection of locally optimized trees that capture regional or source-specific patterns. These local models can subsequently be integrated to create a unified, global set of decision rules.

Decentralized decision tree models have proved effective in data classification for the problem studied in [21]. The methods proposed in [22] used splines and Fisher’s linear discriminant function to generate compact trees in distributed settings, and Monte Carlo Tree Search (MCTS) and its parallel variants [23]. However, it should be noted that in the proposed approach, the decision trees are not tuned in any way and verified regarding the classification accuracy prior to the induction of true general rules.

## 3. Learning General Rules over *S*

This section introduces the main notions and the greedy algorithm used for inducing decision rules from sets of decision trees.

### 3.1. Main Notions

Let *F* be a set of binary attributes and ω be the set of non-negative integers. A decision rule *r* over *F* is expressed in the following form:

$$\left(f_{1}=\delta_{1}\right)\wedge \cdots \wedge \left(f_{m}=\delta_{m}\right)\to d,$$

where m,d∈ω, $f_{1},\ldots ,f_{m}$ are pairwise different attributes from *F*, $\delta_{1},\ldots ,\delta_{m}\in \left\{0,1\right\}$. The left-hand side of this rule is composed of a conjunction of descriptors in the form of $f_{j}=\delta_{j}$. C(r) denotes the set of these descriptors. The right-hand side of *r* is a decision assigned by *d* and denoted as D(r). We indicate that two rules $r_{1}$ and $r_{2}$ are incompatible if there exists an elementary descriptor f=δ such that f=δ belongs to $C(r_{1})$ and f=¬δ belongs to $C(r_{2})$, where ¬0=1 and ¬1=0.

A decision tree over *F* is a marked directed tree with the root in which each terminal node has assigned a decision *d*. Each nonterminal (working) node is labeled with an attribute from *F*. Each working node has two edges exiting from it labeled with the numbers 0 and 1, respectively. In every directed path from the root to a terminal node (also called a complete path), the attributes linked to the working nodes are mutually distinct.

Let Γ be a decision tree. The number of terminal nodes is equal to the number of working nodes plus one. Additionally, the number of complete paths is equal to the number of terminal nodes. Let ξ be a complete path in Γ with *m* working nodes in which the terminal node is labeled with the decision *d*. A decision rule rule(ξ) corresponds to it. If m=0, then this rule is equal to →d. Let m≥1, the working nodes of ξ be labeled with the attributes $f_{1},\ldots ,f_{m}$, and edges leaving these nodes be labeled with the numbers $\delta_{1},\ldots ,\delta_{m}$, respectively. Then, the rule rule(ξ) has the following form:

$$\left(f_{1}=\delta_{1}\right)\wedge \cdots \wedge \left(f_{m}=\delta_{m}\right)\to d.$$

The set of complete paths in Γ is denoted as Ξ(Γ), and the set of decision rules {rule(ξ):ξ∈Ξ(Γ)} corresponding to complete paths in Γ is denoted as IR(Γ). Decision rules from IR(Γ) are called inner decision rules over Γ.

Let *S* represent a finite, nonempty set of decision trees. $IR\left(S\right)={⋃}_{\Gamma \in S}IR\left(\Gamma \right)$, F(S) the set of attributes attached to working nodes of the decision trees from *S*, D(S) the set of decisions attached to terminal nodes of the decision trees from *S*, and GR(S) the set of decision rules over F(S) that have decisions from D(S) in their right-hand sides. IR(S) denotes inner rules over *S*, and GR(S) denotes general rules over *S*, IR(S)⊆GR(S).

Let Γ be a decision tree belonging to the set *S* and *r* be a decision rule from GR(S). The rule *r* is true for Γ if there exists an inner decision rule $r^{\prime }$ over Γ such that $D\left(r^{\prime }\right)=D\left(r\right)$ and $C\left(r^{\prime }\right)\subseteq C\left(r\right)$.

**Remark** **1.** *If such an inner decision rule $r^{\prime }$ over *Γ* exists, then each of the other inner decision rules $r^{\prime \prime }$ over *Γ* and the decision rule r are incompatible.*

### 3.2. Greedy Algorithm

The greedy algorithm is applied iteratively to each decision *d* from the set d∈D(S). The pseudocode is provided in Algorithm 2.
**Algorithm 2** Greedy algorithm for induction of decision rules from the set of decision trees.**Require:** Set *S* of decision trees, decision *d*, set $I_{0}$, and real number α, 0≤α<1.**Ensure:** True decision rule.    $r_{0}$: →d; i=0;    **while** $|I_{0}-I_{i}\left|\geq \right|I_{0}\left||\left(1-\alpha \right)\right|$ **do**        select descriptor f=a that does not belong to the left-hand side of the rule $r_{i}$ and belongs to the maximum number of left-hand sides of rules from $I_{i}$;        $r_{i}⟵r_{i}\cup \left(f=a\right)$;        i=i+1;    **end while**    **return** $r_{i+1}$;

Initially, the set $I_{0}$ consists of all rules from IR(S) where the right-hand side is equal to *d* and the left-hand side contains at least one descriptor. The decision rule has a form →d. At each iteration, an elementary condition f=a that does not belong to the left-hand side of the rule $r_{i}$ and belongs to the maximum number of left-hand sides of rules from $I_{i}$ is selected and added to the left-hand side of the rule $r_{i}$, which is denoted as rule $r_{i+1}$. From the set $I_{i}$, we remove all rules *r* such that *r* and $r_{i+1}$ are incompatible and all rules *r* such that $C\left(r\right)\subseteq C\left(r_{i+1}\right)$. $I_{i+1}$ denotes the set of remaining rules. If $|I_{0}-I_{i}|\geq I_{0}\left|\left(1-\alpha \right)\right|$, then the algorithm terminates.

**Remark** **2.** *By applying Remark 1, it can be demonstrated that for each step i of the greedy algorithm, if the rule $r_{i}$ is true for a decision tree *Γ* from S, then $I_{i}\cap IR\left(\Gamma \right)=\emptyset$.*

**Figure 1:**
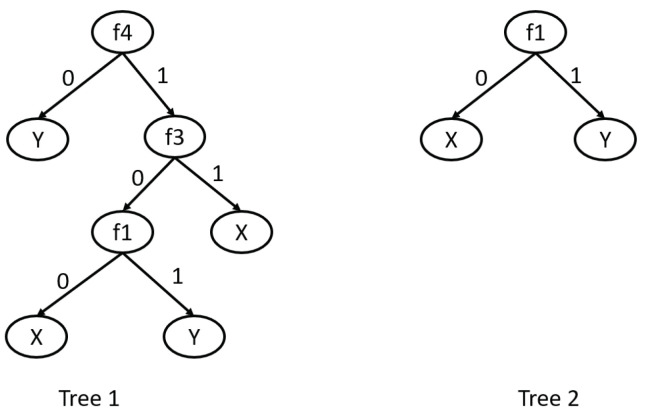
Set of decision trees $S=\{Tree_{1},Tree_{2}\}$.

The example presents the work of the greedy algorithm (Algorithm 2) for the set of decision trees from Figure 1.

The set of inner decision rules is the following: $IR\left(S\right)=\left\{f_{4}=0\to Y,f_{4}=1\wedge f_{3}=0\wedge f_{1}=0\to X,f_{4}=1\wedge f_{3}=0\wedge f_{1}=1\to Y,f_{4}=1\wedge f_{3}=1\to X,f_{1}=0\to X,f_{1}=1\to Y\right\}$. Using α=0.7, the greedy algorithm induces decision rules for each decision class, i.e., X and Y. We start with d=X, so $r_{0}:\to X$. The set $I_{0}$ consists of all rules from IR(S), in which the right-hand side is equal to X and the left-hand side is not empty, so $|I_{0}|=3$. The algorithm works while $|I_{0}-I_{i}\left|\geq \right|I_{0}\left|\left(1-\alpha \right)\right|$, so $|I_{0}-I_{i}|\geq 1$. The algorithm selects condition $f_{1}=0$, which does not belong to the left-hand side of the rule $r_{0}$ and belongs to the maximum number of left-hand sides of rules from $I_{0}$. From the set $I_{i}$, all rules *r* such that *r* and $r_{i+1}$ are incompatible and all rules *r* such that $C\left(r\right)\subseteq C\left(r_{i+1}\right)$ are removed. After this iteration, the obtained rule is the following: $r_{1}:f_{1}=0\to X$ and $I_{1}=\left\{f_{4}=1\wedge f_{3}=0\wedge f_{1}=0\to X,f_{4}=1\wedge f_{3}=1\to X\right\}$, $|I_{0}-I_{i}|\geq 1$, so the stop criterion is satisfied and the obtained rule is true for Tree 2.

In the case where α=0.0, the obtained rule for the decision class X is the following: $f_{1}=0\wedge f_{4}=1\wedge f_{3}=0\to X$. And it is true for 2 decision trees $Tree_{1}$ and $Tree_{2}$ depicted in Figure 1.

## 4. Experimental Results

**Figure 2:**
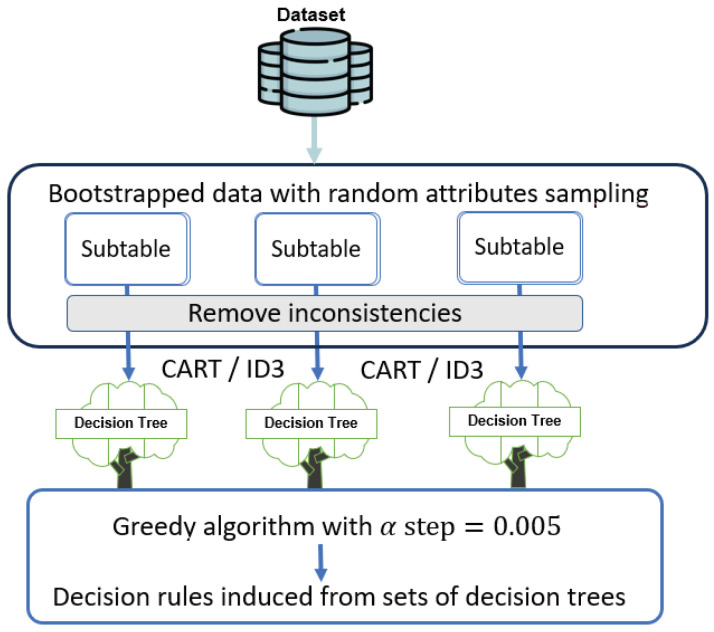
Procedure of experiments.

The research methodology is shown in Figure 2. It comprises the following stages:

1. Data preparation and subtables construction;
2. Induction of decision trees using CART and ID3 algorithms;
3. Induction of decision rules using the greedy algorithm with step α=0.005;
4. Analysis and comparison of obtained results taking into account knowledge representation and performance of the global rule-based classifier perspectives.

Each decision table *T* is divided into subtables using the bootstrapping technique. In this way, random subsets of the original data are generated with a constraint when the minimum number of attributes is 5, and *k* sets of subtables are created, ranging in size from 5 to 10 subtables. These parameters are selected by the authors. Then, inconsistencies are removed by replacing groups of rows with the same values of attributes and different decisions with one row with the most common decision for the group. After that, the data are converted into a binary matrix form using One-Hot Encoding as a technique for transforming categorical attributes into binary form.

Table 1 presents general information about the datasets used during the experiments, i.e., name, number of rows, number of attributes, and number of binary attributes. The datasets come from public repositories, i.e., UCI ML Repository (https://archive.ics.uci.edu/datasets accessed on 1 January 2025)—kr-vs-kp and tic-tac-toe datasets, and Kaggle (https://www.kaggle.com/datasets accessed on 1 January 2025)—customer-churn dataset. Each dataset has two decision classes.

Table 2 presents information about the obtained sets of subtables, which are considered distributed data sources. Rows indicated from 5 to 10 represent the number of subtables in the set. For each dataset, the average number of rows in the set with *m* subtables, m=5,…,10, and the average number of attributes after the One-Hot Encoding procedure are determined. The last row presents the average values.

The highest mean value for the number of attributes is for the kr-vs-kp dataset with 6 subtables, and the lowest is for the tic-tac-toe dataset with 8 subtables. This situation reflects the overall number of attributes and their values for each dataset i.e., kr-vs-kp with the highest value of attributes in the entry column, tic-tac-toe with the lowest value of attributes in the entry column.

For each subtable, a decision tree is generated using the CART and ID3 algorithms. Information about the induced decision trees, their depth, working nodes and terminal nodes is presented in Table 3 as average values. The first column denotes the cardinality of the sets of decision trees. Values in the first row of the table are assigned to the CART algorithm, and those of the second row to the ID3 algorithm. The last row presents average values for both algorithms, respectively.

**Figure 3:**
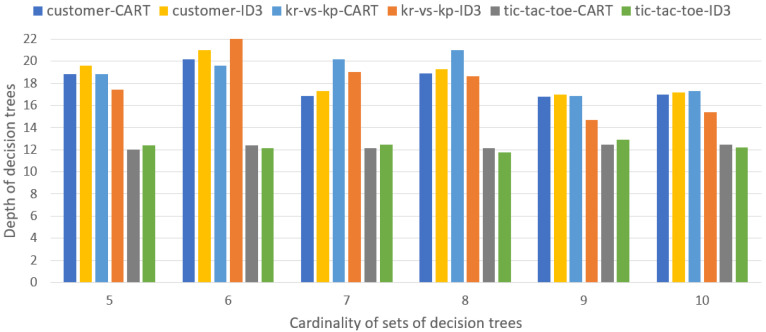
Average depth of decision trees induced from sets with cardinality *m*, m=5,…,10.

Figure 3 presents the average depth of decision trees induced by the CART and ID3 algorithms, for all sets with cardinality *m*, m=5,…,10.

In the case of subtables relating to the customer-churn dataset, the ID3 algorithm generates trees with greater depth than the CART algorithm, but the differences are small. In the case of the kr-vs-kp dataset, the opposite situation occurs, i.e., the depth of the trees induced by the CART algorithm is greater than the depth of the trees induced by the ID3 algorithm, except for the set containing six decision trees. In the case of the tic-tac-toe dataset, the average depth of trees is comparable (with small differences) for both algorithms and all sets of decision trees.

Taking into account the values presented in Table 3, it can be seen that for all sets of decision trees, the lowest depths and lowest number of terminal nodes occur for the tic-tac-toe dataset, while the highest number of terminal nodes occurs for the customer-churn dataset.

**Figure 4:**
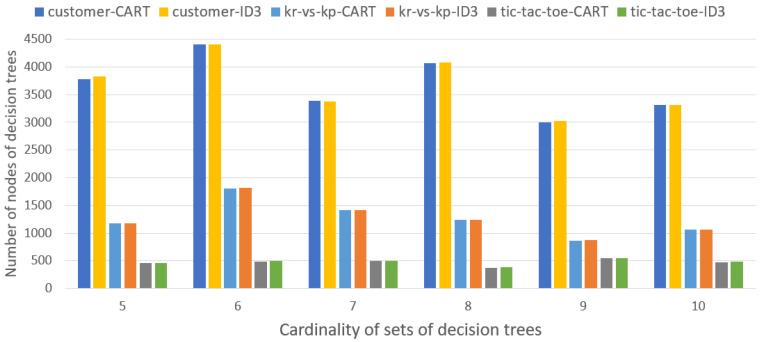
Average number of nodes of decision trees induced from sets with cardinality *m*, m=5,…,10.

Figure 4 presents the average number of nodes of decision trees induced by the CART and ID3 algorithms, for all sets with cardinality *m*, m=5,…,10.

In the general case, the number of nodes (terminal and working) in the decision trees induced by the CART and ID3 algorithms is comparable for all sets of decision trees.

For the induction of decision rules from a set of decision trees, the greedy algorithm (see Algorithm 2) is applied, with increasing the value of the α parameter as step α=0.005, for all sets of decision trees with *m* cardinality, m=5,…,10.

Table 4, Table 5 and Table 6 show the length of rules that are true for the maximum number of trees from a set of *m* decision tress, m=5,…,10 (column len) and the number of trees for which the rules are true (column tree). The first row of the table concerns trees induced by the CART algorithm, and the second row concerns trees induced by the ID3 algorithm. The α values are incremented with a step 0.005, and columns of the table show the values of minimum α for which there is a change in the number of trees for which the rules are true. Value 0 in the column tree indicates that there are no trees from the set of trees for which rules are true (denoted with the grey color in the column len). Where there is more than one rule true for the maximum number of trees from the set, the values in the column len are bolded. The last column of the table denotes the maximum value of α for which there exists at least one tree for which the rule is true. The last row presents the average values for the CART and ID3 algorithms, respectively.

The presented results show that for the considered datasets and sets of decision trees, the constructed rules are relatively long for α=0.0. The largest rule lengths are observed for the kr-vs-kp set and the set containing 10 decision trees. It is worth noting that in Table 5 (the set of seven decision trees induced using the CART algorithm) and Table 4 (the set of six decision trees induced using the CART and ID3 algorithms and the set of eight decision trees induced using the CART algorithm), there are two rules that are true for the maximum number of trees. The length of the induced rules decreases as the α value increases, and the number of trees for which the rules are true also varies. These values are different and depend on the dataset and the distribution of attribute values. For example, for the set tic-tac-toe and α = 0.015, all constructed rules are true for at least one decision tree from the set of decision trees, but in the case of the customer and kr-vs-kp datasets, there is unfortunately a small number of cases where the constructed rules are true for at least one decision tree from the set of decision trees. This indicates the need for future studies to consider a smaller α step when working with the greedy algorithm. The induced rules are relatively long, but it should be taken into account that the general rule optimization problem is NP-hard, and the obtained rules should allow to properly classify new cases in a distributed environment.

The Friedman test [24] is performed to provide insight into the algorithm’s behavior for different values of α for the length of the rules and the number of trees for which the rules are true. A common range of α values (0.0,0.005,0.01,0.015,0.02) is selected for comparison across all datasets for the length of the rules and the number of trees for which the rules are true. This range is derived by analyzing distributed sources, with values averaged to ensure consistency and comparability. Each α value reflects the performance of decision rules aggregated from subsets of the distributed data, ensuring that the results represent a unified view of the algorithm’s behavior. Both the rule length and the number of trees for which the rules are true following Friedman’s test gives a *p*-value of 0.017 for both CART and ID3. This suggests that there is a significant change in the length of the rules, as well as in the number of trees for which the rules are true, when α is varied.

**Figure 5:**
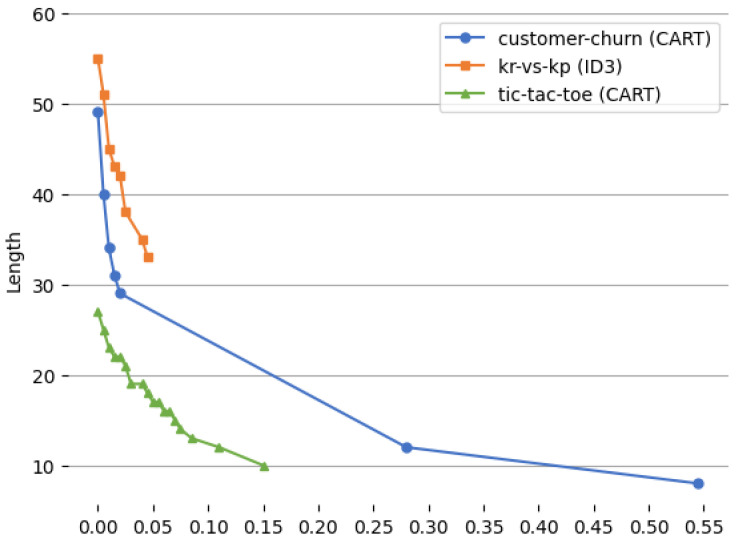
Relationship between the values of the α parameter and the lengths of the induced decision rules.

**Figure 6:**
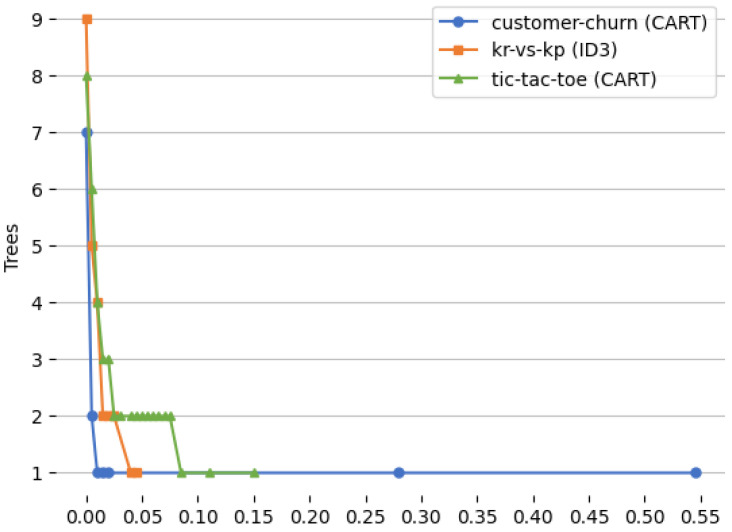
Relationship between the values of the α parameter and the number of decision trees for which the induced decision rules are true.

Figure 5 and Figure 6 show the relationship between the values of the α parameter and the lengths of the induced rules and between the values of the α parameter and the number of trees for which the rules are true, respectively.

The charts (Figure 5 and Figure 6) show the effect of the changes in the values of α from zero till the maximum α for which the number of trees that are true is minimal. As the parameter α increases from zero, a distinct effect can be observed on both the length of the extracted rules and the number of trees in the ensemble for which these rules are true. At low values of α, the algorithm favors complex, detailed rules that accurately capture the nuances of the data, resulting in longer rule lengths. In this scenario, the extracted rules are typically true across a larger number of trees, reflecting a higher number of trees for which the rules are true. However, as α increases, the algorithm begins to prioritize simplicity, leading to shorter rules that contain fewer conditions. This reduction in rule length suggests that the algorithm is increasingly willing to tolerate a slight loss in generalization in exchange for interpretability and conciseness. Consequently, fewer trees are required to satisfy these simpler rules, as they are more generalized and less tailored to specific data patterns. Intuitively, this progression shows that higher values of α encourage the algorithm to streamline the model, producing rules that are easier to interpret and apply, albeit at the cost of some specificity as well as losing generalization. This trade-off, controlled by alpha can be used to tune the model.

The results presented so far pertain to data analysis from the perspective of knowledge representation. It is evident that a crucial aspect of the research involves the predictive capabilities of the constructed data model. For this purpose, each subset in the dataset of size *m*, m=5,…,10 is divided into a training part (70%) and a testing part (30%). Based on the training parts of subtables, decision trees were induced using the CART and ID3 algorithms. Next, for each set of *m* decision trees, where m=5,…,10, a greedy algorithm was applied with the parameter α, incrementing α by 0.005, from 0 to 0.2. The decision rule sets obtained in this way are then applied to the test parts of the subtables within the set of size *m*. Classification accuracy is defined as the ratio of misclassified objects in the test subtable to the total number of objects in the test subtable. Table 7 shows the average values of classification accuracy derived from the number of subtables within the dataset of size *m* (column acc) and the standard deviation (column std). The values in the first row relate to the results associated with decision trees induced by the CART algorithm, and in the second row, to those induced by the ID3 algorithm. Sign “-” in cells of the tables denotes that there are no true rules induced by the greedy algorithm.

The results presented in Table 7 refer to α values from 0 to 0.01 (for kr-vs-kp and tic-tac-toe) since for higher values of this parameter, for the considered datasets, the number of true rules for the set of decision trees decreases but only the true rules are taken into account in the construction of the classification model. This situation arises, for example, for kr-vs-kp and a set of 6 decision trees induced using the ID3 algorithm. For most cases, as α increases, the classification accuracy increases slightly or remains the same as that for α=0.0 level. In the case of the tic-tac-toe dataset and α=0.005, a decrease in classification quality can be observed in five cases. The Wilcoxon test conducted shows that there is no statistically significant difference in the averages of the paired accuracies of the CART and ID3 algorithms. Given the very small number of rules constituting the classifier and the distributed data sources environment on which the model is tested, the proposed greedy algorithm provides the possibility to learn decision rules from ensembles of decision trees for knowledge discovery.

## 5. Conclusions

The advancement of distributed technologies and the growing volume of data from diverse sources necessitate the development of algorithms for distributed data analysis. This paper investigates the problem of learning general decision rules that are true for a set of decision trees. General rules are arbitrary rules that use attributes from the considered decision trees.

The main contribution of this work lies in the development of a research methodology for inducing decision rules from a set of decision trees. A novelty is the introduction of a greedy algorithm designed to derive decision rules from distributed data sources. This algorithm incorporates an α parameter to construct approximate decision rules, striking a balance between rule accuracy and length. The resulting decision rules, derived from decision trees representing distributed knowledge sources, can be considered a global rule-based model that encapsulates the main patterns within the data. A notable application of this approach is in the medical field, where patients’ data from various facilities can be unified to create a global model for example, for diagnostic procedures. This model has the potential to streamline diagnosis and treatment processes, leading to improved patient care.

The study was carried out on randomly constructed subtables included in sets with *m* cardinality, m=5,…,10. Decision trees were induced using traditional algorithms, such as CART and ID3. The greedy algorithm was applied with different values of the α parameter. Tests have shown that as the α value increases, the length of the induced rules decreases, as does the number of trees for which the rules are true. For most of the cases analyzed, an increase in the value of α did not lead to a decrease in classification quality. An important aspect of the created rule-based model is the very small number of rules. Their length depends on the characteristics of the dataset and the distribution of attribute values. The conducted studies did not reveal also significant differences in the structure of trees created using the ID3 and CART algorithms.

Future research will focus on modifying the greedy strategy to reduce the number of descriptors forming decision rules. Additionally, methods for ranking attributes and their application in the operation of the greedy algorithm will be analyzed. Currently, the number of rules constructed is related to the number of decision classes; in the future, the possibility of increasing the number of rules forming the model will be explored. The experimental results revealed that the most significant differences in the lengths of decision rules occurred for α values ranging from 0 to 0.02. Therefore, in future studies, this range of α values will be analyzed in the context of new strategies for selecting descriptors used to construct decision rules.

## Figures and Tables

**Table 1:** Description of datasets.

Dataset	Rows	Attributes	Binary Attributes
customer-churn	5794	20	54
kr-vs-kp	3196	37	72
tic-tac-toe	958	10	27

**Table 2:** Description of sets of subtables.

	Customer		kr-vs-kp		tic-tac-toe	
	Rows	Attr	Rows	Attr	Rows	Attr
5	2975.4	42.4	996.2	45.6	424.8	19.8
6	3425.2	46.7	1457.0	60.3	430.8	20.5
7	2455.3	33.1	1158.1	50.1	470.0	21.9
8	3072.6	40.3	1039.1	47.3	374.3	19.1
9	2412.9	37.4	707.8	38.8	504.0	22.7
10	2559.4	37.9	886.0	42.9	426.2	20.4
avg	2816.8	39.6	1040.7	47.5	438.4	20.7

**Table 3:** Characteristics of sets of decision trees.

	Customer			kr-vs-kp			tic-tac-toe		
	**Depth**	**Nodes**	**Terminal**	**Depth**	**Nodes**	**Terminal**	**Depth**	**Nodes**	**Terminal**
5	18.8	2519.0	1260.0	16.8	787.8	394.4	12.0	307.0	154.0
	19.6	2550.2	1275.6	17.4	785.0	393.0	12.4	304.6	152.8
6	20.2	2935.0	1468.0	21.2	1200.0	600.5	12.2	324.7	162.8
	21.0	2938.7	1469.8	22.0	1213.3	607.2	12.2	334.7	167.8
7	16.9	2257.6	1129.3	18.4	946.1	473.6	12.4	327.0	164.0
	17.3	2254.1	1127.6	19.0	941.9	471.4	12.4	335.0	168.0
8	18.9	2710.8	1355.9	17.5	821.8	411.4	11.5	246.8	123.9
	19.3	2720.5	1360.8	18.6	823.5	412.3	11.8	257.5	129.3
9	16.8	1999.9	1000.4	14.7	577.0	289.0	12.7	361.0	181.0
	17.0	2019.4	1010.2	14.7	581.9	291.4	12.9	361.7	181.3
10	17.0	2209.4	1105.2	15.5	711.6	356.3	12.1	311.2	156.1
	17.2	2211.6	1106.3	15.4	709.4	355.2	12.2	320.2	160.6
avg	18.1	2438.6	1219.8	17.4	840.7	420.9	12.2	313.0	157.0
	18.6	2449.1	1225.1	17.9	842.5	421.8	12.3	319.0	160.0

**Table 4:** Characteristics of decision rules induced from sets of decision trees for the customer-churn dataset.

	α=0.0		α=0.005		α=0.01		α=0.015		α=0.02		α=0.28		α=0.545	
	**Len**	**Tree**	**Len**	**Tree**	**Len**	**Tree**	**Len**	**Tree**	**Len**	**Tree**	**Len**	**Tree**	**Len**	**Tree**
5	49.0	5	36.0	1	33.0	0	30.5	0	28.0	0	10.0	0	4.5	0
	48.0	4	38.0	2	33.0	1	31.0	0	29.5	0	11.0	0	6.5	0
6	**48.5**	5	38.0	0	31.5	0	29.5	0	27.0	0	10.0	0	5.5	0
	**47.5**	5	35.0	0	30.5	0	28.0	0	26.5	0	11.0	0	7.0	0
7	50.0	6	39.0	2	35.0	2	32.0	1	28.5	0	11.5	0	6.0	0
	53.0	7	38.5	0	32.5	0	29.0	0	27.5	0	12.0	0	8.0	0
8	**48.5**	5	35.0	1	31.0	1	28.5	0	27.0	0	11.5	0	6.0	0
	51.0	7	37.5	0	32.5	0	29.5	0	27.5	0	12.5	0	6.0	0
9	49.0	7	40.0	2	34.0	1	31.0	1	29.0	1	12.0	1	8.0	1
	48.0	6	39.5	0	32.5	0	29.5	0	28.0	0	12.0	0	7.5	0
10	51.0	10	38.0	1	32.5	0	30.5	0	28.0	0	13.0	0	8.0	0
	51.0	7	42.0	1	36.0	1	33.0	1	31.0	1	13.0	0	8.0	0
avg	49.3	6.3	37.7	1.2	32.8	0.7	30.3	0.3	27.9	0.2	11.3	0.2	6.3	0.2
	49.8	6.0	38.4	0.5	32.8	0.3	30.0	0.2	28.3	0.2	11.9	0.0	7.2	0.0

**Table 5:** Characteristics of decision rules induced from sets of decision trees for the kr-vs-kp dataset.

	α=0.0		α=0.005		α=0.01		α=0.015		α=0.02		α=0.025		α=0.04		α=0.045	
	**Len**	**Tree**	**Len**	**Tree**	**Len**	**Tree**	**Len**	**Tree**	**Len**	**Tree**	**Len**	**Tree**	**Len**	**Tree**	**Len**	**Tree**
5	51.0	5	48.0	4	42.0	2	39.0	1	32.5	0	30.0	0	24.0	0	23.5	0
	50.0	5	45.0	3	44.0	1	39.0	1	36.0	1	35.0	1	29.0	1	29.0	1
6	56.0	5	49.5	0	43.5	0	40.5	0	37.5	0	35.5	0	32.5	0	30.5	0
	57.0	6	50.0	2	47.0	1	41.5	0	39.5	0	38.0	0	30.5	0	29.5	0
7	**61.0**	6	55.0	4	52.0	2	46.5	0	42.5	0	39.0	0	36.0	0	33.5	0
	56.0	7	49.0	4	45.0	1	41.0	1	37.0	0	36.0	0	30.0	0	28.5	0
8	60.0	8	54.0	3	49.0	2	44.0	1	36.0	0	33.5	0	28.5	0	26.5	0
	61.0	7	57.0	3	51.0	1	43.5	0	40.5	0	37.5	0	31.0	0	28.0	0
9	55.0	9	51.0	6	46.0	4	43.0	2	40.0	2	36.0	1	30.0	0	29.5	0
	55.0	9	51.0	5	45.0	4	43.0	2	42.0	2	38.0	2	35.0	1	33.0	1
10	66.0	10	60.0	5	51.0	2	42.5	0	38.5	0	37.5	0	31.5	0	30.0	0
	61.0	10	59.0	5	49.0	2	45.0	2	41.0	1	35.0	0	31.0	0	28.5	0
avg	58.2	7.2	52.9	3.7	47.3	2.0	42.6	0.7	37.8	0.3	35.3	0.2	30.4	0.0	28.9	0.0
	56.7	7.3	51.8	3.7	46.8	1.7	42.2	1.0	39.3	0.7	36.6	0.5	31.1	0.3	29.4	0.3

**Table 6:** Characteristics of decision rules induced from sets of decision trees for the tic-tac-toe dataset.

	α=0.0		α=0.005		α=0.01		α=0.015		α=0.02		α=0.025		α=0.03		α=0.04		α=0.045	
	**Len**	**Tree**	**Len**	**Tree**	**Len**	**Tree**	**Len**	**Tree**	**Len**	**Tree**	**Len**	**Tree**	**Len**	**Tree**	**Len**	**Tree**	**Len**	**Tree**
5	19.0	5	18.0	4	17.0	3	16.0	2	16.0	2	15.0	1	14.0	1	13.0	1	12.0	1
	23.0	5	23.0	5	22.0	3	19.0	2	19.0	2	16.0	0	15.5	0	13.5	0	13.0	0
6	25.0	6	25.0	6	24.0	4	22.0	4	21.0	3	19.0	3	18.0	1	17.0	1	15.0	0
	26.0	5	24.0	4	23.0	2	22.0	2	20.5	1	20.0	1	20.0	1	18.0	1	18.0	1
7	**25.5**	4	23.5	3	21.5	2	19.0	2	19.0	2	19.0	2	18.0	2	17.0	2	17.0	2
	27.0	7	25.0	5	24.0	4	22.0	2	21.0	2	20.0	2	20.0	2	18.0	2	18.0	2
8	27.0	8	25.0	6	23.0	4	22.0	3	22.0	3	21.0	2	19.0	2	19.0	2	18.0	2
	25.0	5	24.5	3	23.5	2	22.0	2	21.0	2	20.0	2	20.0	2	19.0	2	18.0	1
9	27.0	8	26.0	7	25.0	5	24.0	4	21.0	3	20.0	1	19.0	1	16.0	1	15.0	1
	26.0	9	26.0	9	25.0	6	22.0	5	19.0	5	18.0	4	17.0	4	15.0	1	14.5	1
10	27.0	8	27.0	8	24.0	3	21.0	1	21.0	1	20.0	1	19.0	1	17.0	1	17.0	1
	26.0	7	24.5	4	23.0	2	22.0	1	20.0	1	20.0	1	19.5	0	17.0	0	17.0	0
avg	25.1	6.5	24.1	5.7	22.4	3.5	20.7	2.7	20.0	2.3	19.0	1.7	17.8	1.3	16.5	1.3	15.7	1.2
	25.5	6.3	24.5	5.0	23.4	3.2	21.5	2.3	20.1	2.2	19.0	1.7	18.7	1.5	16.8	1.0	16.4	0.8
	α=0.05		α=0.055		α=0.06		α=0.065		α=0.07		α=0.075		α=0.085		α=0.11		α=0.15	
	**len**	**tree**	**len**	**tree**	**len**	**tree**	**len**	**tree**	**len**	**tree**	**len**	**tree**	**len**	**tree**	**len**	**tree**	**len**	**tree**
5	11.0	1	11.0	1	11.0	1	11.0	1	10.0	0	10.0	0	9.0	0	8.0	0	7.0	0
	12.5	0	12.0	0	11.5	0	11.5	0	11.0	0	10.5	0	9.5	0	8.0	0	7.5	0
6	15.0	0	13.5	0	13.5	0	13.0	0	12.0	0	12.0	0	11.0	0	8.5	0	7.5	0
	17.0	1	17.0	1	13.5	0	13.0	0	12.5	0	12.5	0	12.0	0	9.5	0	8.0	0
7	16.0	1	16.0	1	16.0	1	14.0	0	13.5	0	13.0	0	11.5	0	10.5	0	9.5	0
	17.0	2	16.0	1	16.0	1	15.0	1	15.0	1	15.0	1	14.0	1	11.5	0	9.5	0
8	17.0	2	17.0	2	16.0	2	16.0	2	15.0	2	14.0	2	13.0	1	12.0	1	10.0	1
	18.0	1	17.0	1	17.0	1	16.0	1	15.0	1	14.0	0	13.0	0	12.5	0	10.0	0
9	14.0	1	14.0	1	14.0	1	14.0	1	13.0	1	13.0	1	13.0	1	9.0	1	7.0	0
	15.0	1	15.0	1	14.0	1	14.0	1	12.5	0	12.0	0	12.0	0	10.0	0	8.5	0
10	16.0	1	15.0	1	14.0	1	14.0	1	14.0	1	12.5	0	12.0	0	10.5	0	9.0	0
	16.0	0	15.5	0	14.5	0	14.5	0	14.0	0	13.0	0	12.5	0	11.5	0	10.0	0
avg	14.8	1.0	14.4	1.0	14.1	1.0	13.7	0.8	12.9	0.7	12.4	0.5	11.6	0.3	9.8	0.3	8.3	0.2
	15.9	0.8	15.4	0.7	14.4	0.5	14.0	0.5	13.3	0.3	12.8	0.2	12.2	0.2	10.5	0.0	8.9	0.0

**Table 7:** Classification accuracy of decision rules induced from sets of decision trees for the customer-churn, kr-vs-kp and tic-tac-toe datasets.

	Customer				kr-vs-kp						tic-tac-toe					
	α=0.0		α=0.005		α=0.0		α=0.005		α=0.01		α=0.0		α=0.005		α=0.01	
	**acc**	**std**	**acc**	**std**	**acc**	**std**	**acc**	**std**	**acc**	**std**	**acc**	**std**	**acc**	**std**	**acc**	**std**
5	0.612	0.174	-	-	0.699	0.176	0.699	0.176	0.699	0.176	0.721	0.113	0.721	0.113	0.721	0.113
	0.388	0.174	0.388	0.174	0.699	0.176	0.699	0.176	0.699	0.176	0.721	0.113	0.721	0.113	0.721	0.113
6	0.479	0.042	0.495	0.010	0.637	0.106	0.637	0.106	0.637	0.106	0.745	0.112	0.745	0.112	0.745	0.112
	0.479	0.042	0.479	0.042	0.547	0.047	0.637	0.106	-	-	0.581	0.053	0.255	0.112	0.255	0.112
7	0.545	0.050	0.671	0.154	0.664	0.141	0.664	0.141	0.664	0.141	0.756	0.091	0.756	0.091	0.756	0.091
	0.671	0.154	0.671	0.154	0.451	0.056	0.664	0.141	0.664	0.141	0.417	0.040	0.400	0.070	0.756	0.091
8	0.580	0.146	0.580	0.146	0.694	0.135	0.694	0.135	0.694	0.135	0.789	0.113	0.211	0.113	0.789	0.113
	0.464	0.066	-	-	0.306	0.135	0.551	0.030	0.694	0.135	0.789	0.113	0.789	0.113	0.789	0.113
9	0.684	0.182	0.684	0.182	0.788	0.145	0.788	0.145	0.788	0.145	0.436	0.066	0.267	0.089	0.267	0.089
	0.684	0.182	0.684	0.182	0.788	0.145	0.788	0.145	0.788	0.145	0.267	0.089	0.267	0.089	0.267	0.089
10	0.446	0.062	0.455	0.034	0.745	0.154	0.745	0.154	0.745	0.154	0.433	0.043	0.692	0.048	0.692	0.048
	0.459	0.046	0.656	0.185	0.745	0.154	0.745	0.154	0.745	0.154	0.692	0.048	0.692	0.048	0.447	0.046
avg	0.558	0.109	0.577	0.105	0.705	0.143	0.705	0.143	0.705	0.143	0.647	0.090	0.565	0.094	0.662	0.094
	0.524	0.111	0.576	0.147	0.589	0.119	0.681	0.125	0.718	0.150	0.578	0.076	0.521	0.091	0.539	0.094

## Data Availability

Datasets used during the experiments are downloaded from UCI Machine Learning Repository https://archive.ics.uci.edu and Kaggle repository https://www.kaggle.com/datasets (accessed on 2 September 2024).
